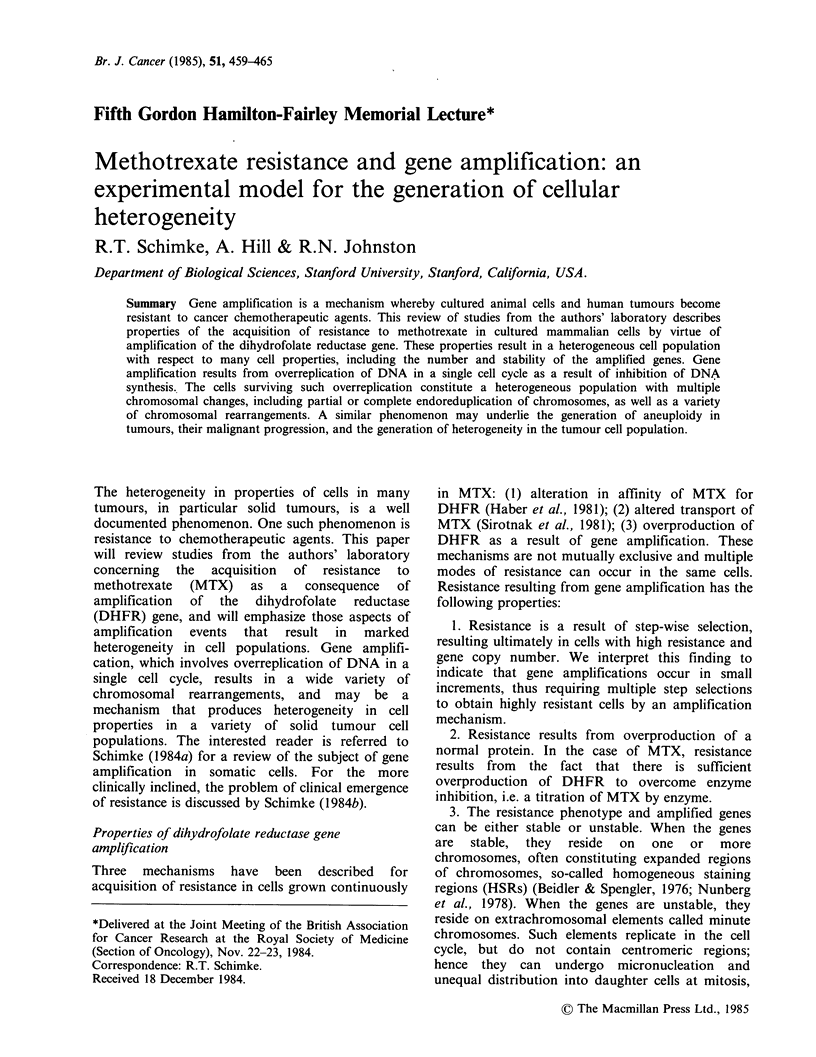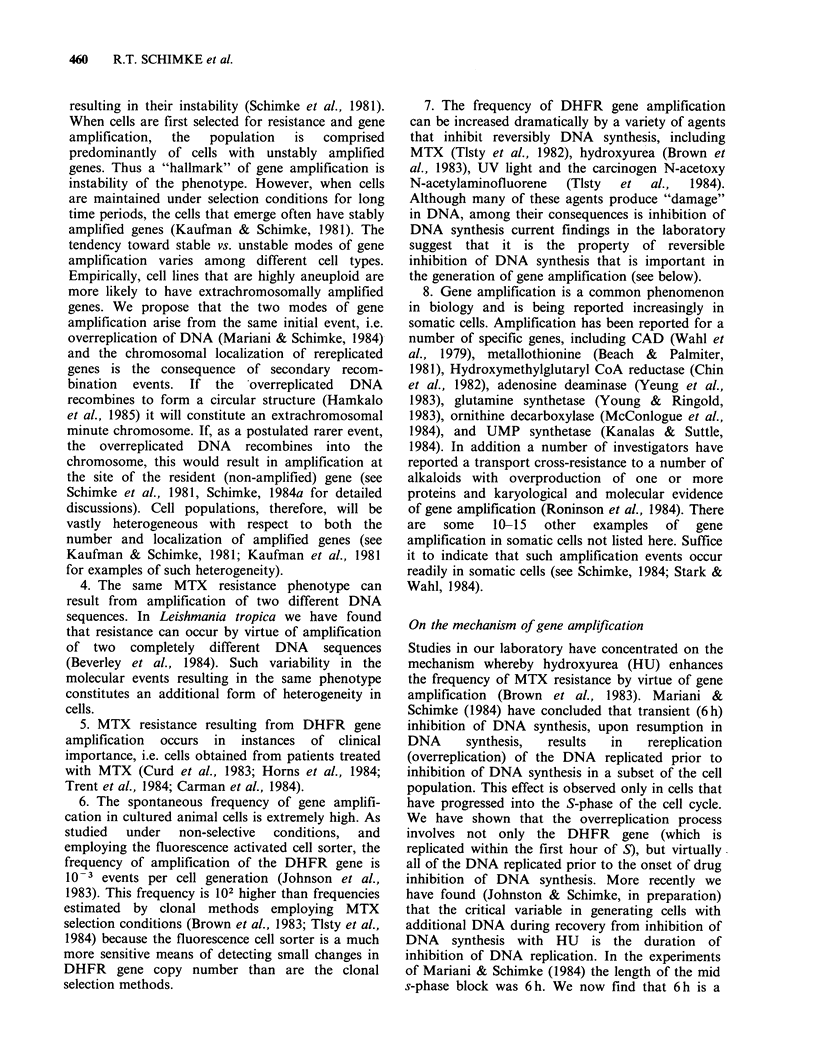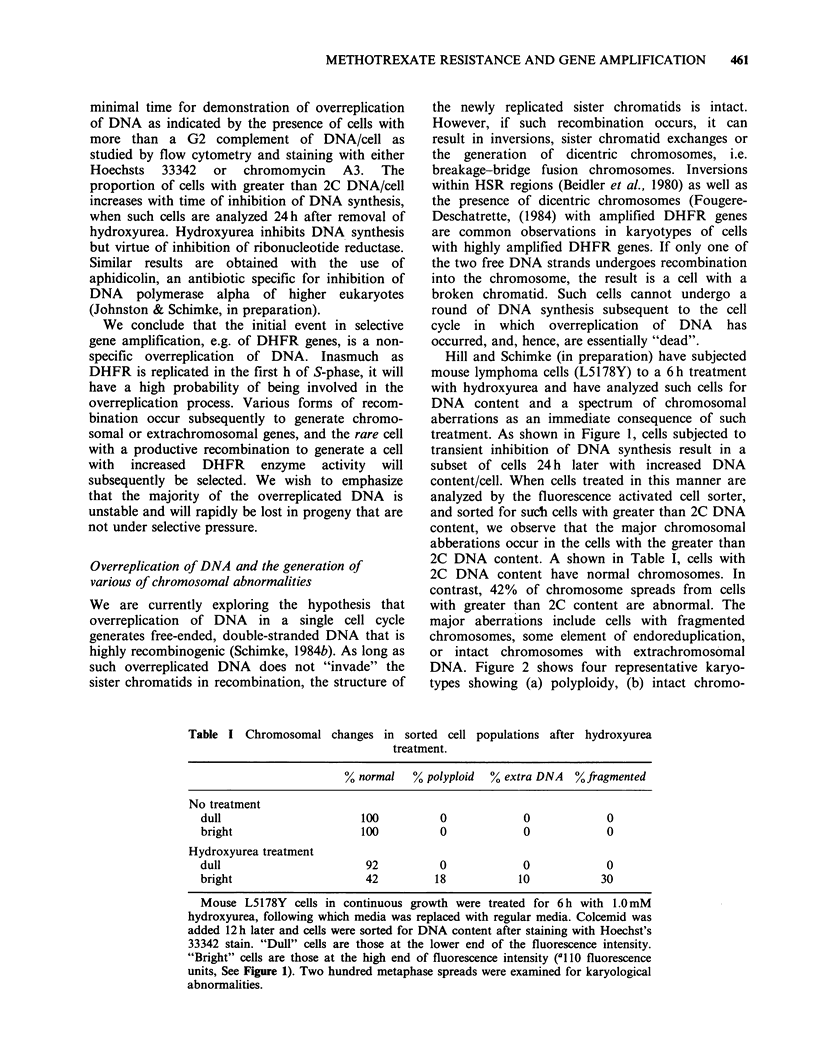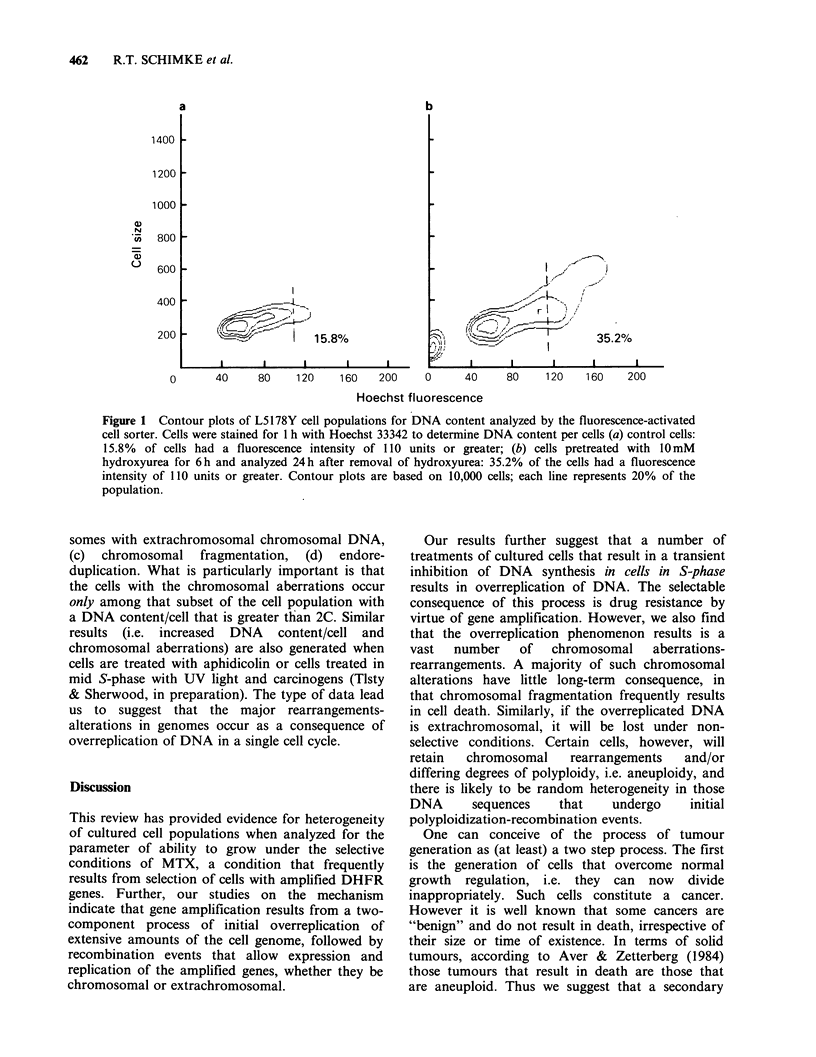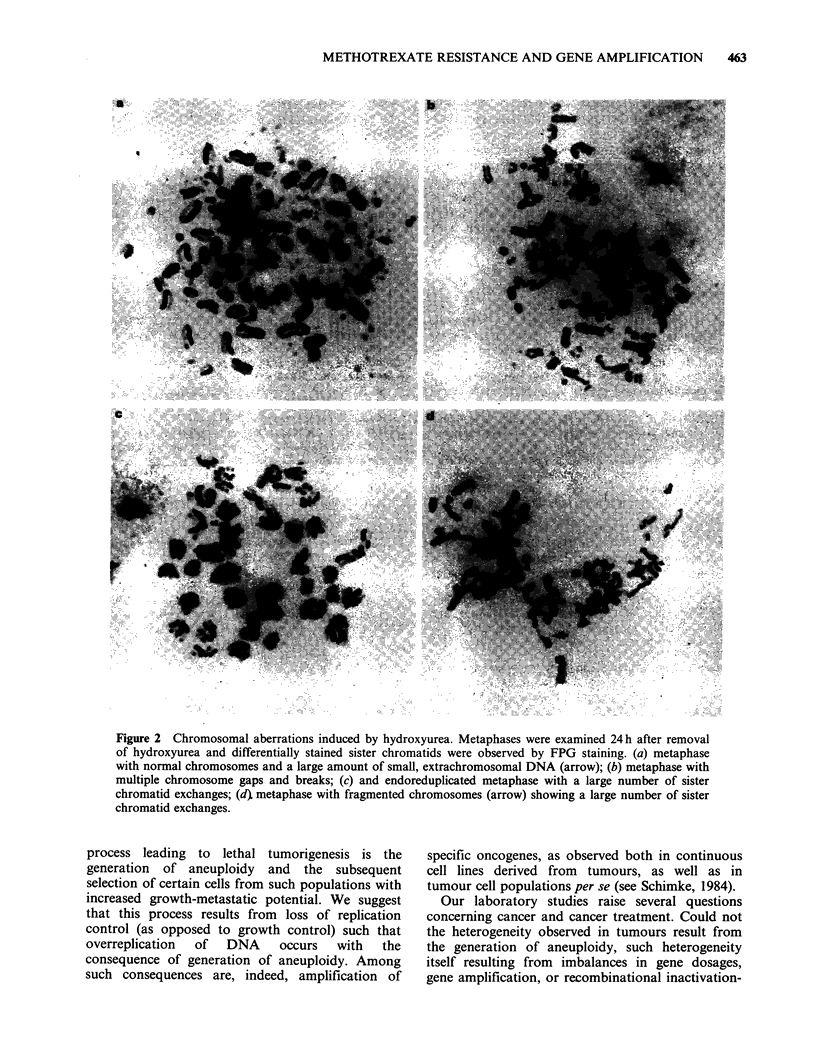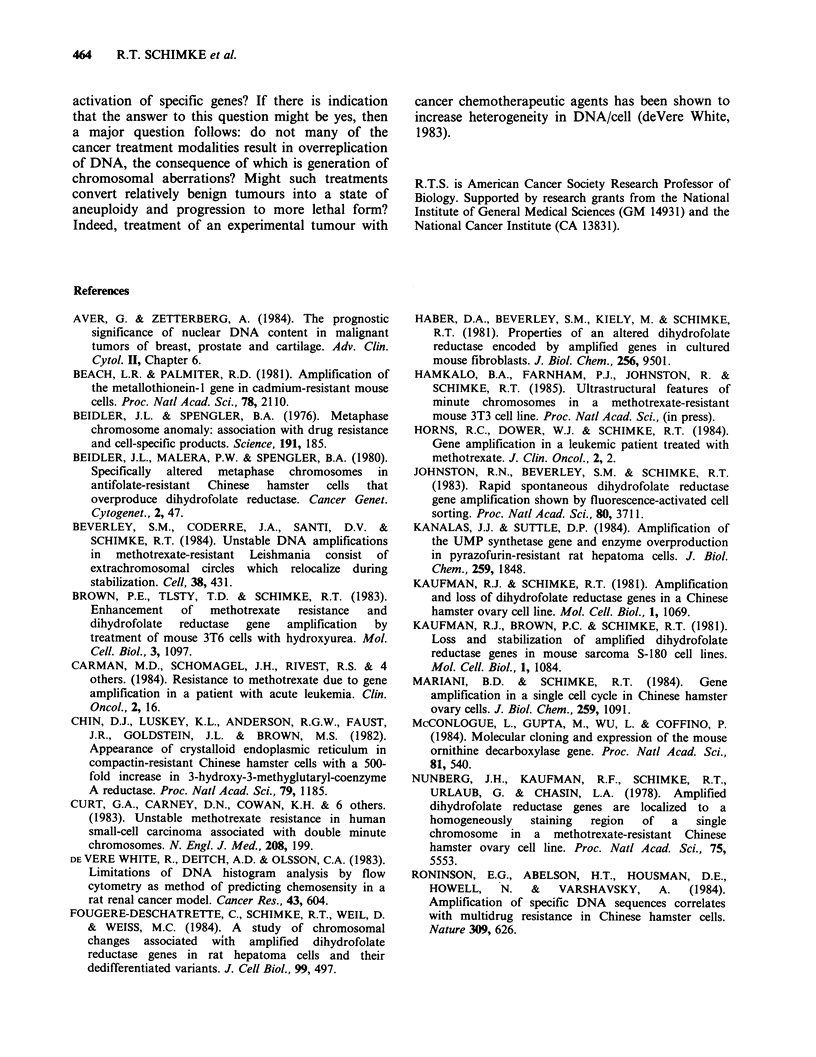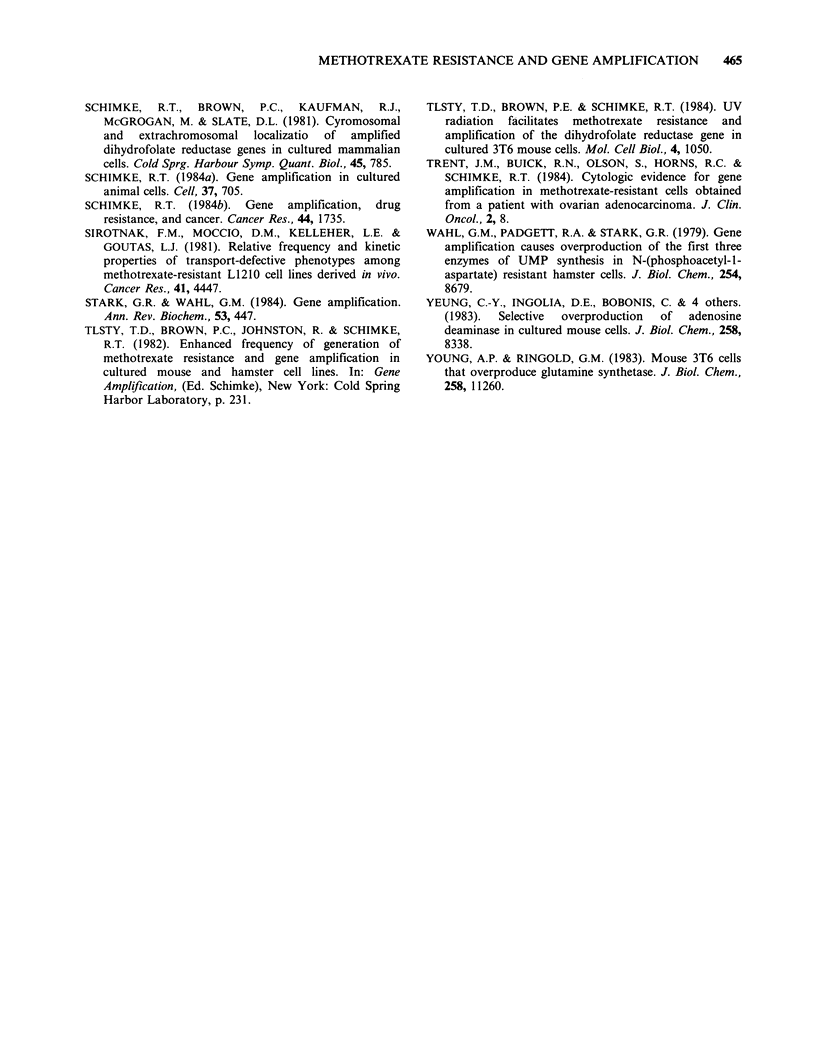# Fifth Gordon Hamilton-Fairley memorial lecture. Methotrexate resistance and gene amplification: an experimental model for the generation of cellular heterogeneity.

**DOI:** 10.1038/bjc.1985.66

**Published:** 1985-04

**Authors:** R. T. Schimke, A. Hill, R. N. Johnston

## Abstract

**Images:**


Br. J. Cancer (1985), 51, 459–465

### Fifth Gordon Hamilton-Fairley Memorial Lecture*

### Methotrexate resistance and gene amplification: an experimental model for the generation of cellular heterogeneity

R.T. Schimke, A. Hill & R.N. Johnston

*Department of Biological Sciences, Stanford University, Stanford, California, USA.*

**Summary** Gene amplification is a mechanism whereby cultured animal cells and human tumours become resistant to cancer chemotherapeutic agents. This review of studies from the authors' laboratory describes properties of the acquisition of resistance to methotrexate in cultured mammalian cells by virtue of amplification of the dihydrofolate reductase gene. These properties result in a heterogeneous cell population with respect to many cell properties, including the number and stability of the amplified genes. Gene amplification results from overreplication of DNA in a single cell cycle as a result of inhibition of DNA synthesis. The cells surviving such overreplication constitute a heterogeneous population with multiple chromosomal changes, including partial or complete endoreduplication of chromosomes, as well as a variety of chromosomal rearrangements. A similar phenomenon may underlie the generation of aneuploidy in tumours, their malignant progression, and the generation of heterogeneity in the tumour cell population.

The heterogeneity in properties of cells in many tumours, in particular solid tumours, is a well documented phenomenon. One such phenomenon is resistance to chemotherapeutic agents. This paper will review studies from the authors' laboratory concerning the acquisition of resistance to methotrexate (MTX) as a consequence of amplification of the dihydrofolate reductase (DHFR) gene, and will emphasize those aspects of amplification events that result in marked heterogeneity in cell populations. Gene amplification, which involves overreplication of DNA in a single cell cycle, results in a wide variety of chromosomal rearrangements, and may be a mechanism that produces heterogeneity in cell properties in a variety of solid tumour cell populations. The interested reader is referred to Schimke (1984*a*) for a review of the subject of gene amplification in somatic cells. For the more clinically inclined, the problem of clinical emergence of resistance is discussed by Schimke (1984*b*).

### *Properties of dihydrofolate reductase gene amplification*

Three mechanisms have been described for acquisition of resistance in cells grown continuously in MTX: (1) alteration in affinity of MTX for DHFR (Haber *et al.*, 1981); (2) altered transport of MTX (Sirotnak *et al.*, 1981); (3) overproduction of DHFR as a result of gene amplification. These mechanisms are not mutually exclusive and multiple modes of resistance can occur in the same cells. Resistance resulting from gene amplification has the following properties:

1. Resistance is a result of step-wise selection, resulting ultimately in cells with high resistance and gene copy number. We interpret this finding to indicate that gene amplifications occur in small increments, thus requiring multiple step selections to obtain highly resistant cells by an amplification mechanism.
2. Resistance results from overproduction of a normal protein. In the case of MTX, resistance results from the fact that there is sufficient overproduction of DHFR to overcome enzyme inhibition, i.e. a titration of MTX by enzyme.
3. The resistance phenotype and amplified genes can be either stable or unstable. When the genes are stable, they reside on one or more chromosomes, often constituting expanded regions of chromosomes, so-called homogeneous staining regions (HSRs) (Beidler & Spengler, 1976; Nunberg *et al.*, 1978). When the genes are unstable, they reside on extrachromosomal elements called minute chromosomes. Such elements replicate in the cell cycle, but do not contain centromeric regions; hence they can undergo micronucleation and unequal distribution into daughter cells at mitosis,

*Delivered at the Joint Meeting of the British Association for Cancer Research at the Royal Society of Medicine (Section of Oncology), Nov. 22–23, 1984.
Correspondence: R.T. Schimke.
Received 18 December 1984.

© The Macmillan Press Ltd., 1985

resulting in their instability (Schimke *et al.*, 1981). When cells are first selected for resistance and gene amplification, the population is comprised predominantly of cells with unstably amplified genes. Thus a "hallmark" of gene amplification is instability of the phenotype. However, when cells are maintained under selection conditions for long time periods, the cells that emerge often have stably amplified genes (Kaufman & Schimke, 1981). The tendency toward stable *vs.* unstable modes of gene amplification varies among different cell types. Empirically, cell lines that are highly aneuploid are more likely to have extrachromosomally amplified genes. We propose that the two modes of gene amplification arise from the same initial event, i.e. overreplication of DNA (Mariani & Schimke, 1984) and the chromosomal localization of rereplicated genes is the consequence of secondary recombination events. If the overreplicated DNA recombines to form a circular structure (Hamkalo *et al.*, 1985) it will constitute an extrachromosomal minute chromosome. If, as a postulated rarer event, the overreplicated DNA recombines into the chromosome, this would result in amplification at the site of the resident (non-amplified) gene (see Schimke *et al.*, 1981, Schimke, 1984*a* for detailed discussions). Cell populations, therefore, will be vastly heterogeneous with respect to both the number and localization of amplified genes (see Kaufman & Schimke, 1981; Kaufman *et al.*, 1981 for examples of such heterogeneity).

4. The same MTX resistance phenotype can result from amplification of two different DNA sequences. In *Leishmania tropica* we have found that resistance can occur by virtue of amplification of two completely different DNA sequences (Beverley *et al.*, 1984). Such variability in the molecular events resulting in the same phenotype constitutes an additional form of heterogeneity in cells.

5. MTX resistance resulting from DHFR gene amplification occurs in instances of clinical importance, i.e. cells obtained from patients treated with MTX (Curd *et al.*, 1983; Horns *et al.*, 1984; Trent *et al.*, 1984; Carman *et al.*, 1984).

6. The spontaneous frequency of gene amplification in cultured animal cells is extremely high. As studied under non-selective conditions, and employing the fluorescence activated cell sorter, the frequency of amplification of the DHFR gene is $10^{-3}$ events per cell generation (Johnson *et al.*, 1983). This frequency is $10^2$ higher than frequencies estimated by clonal methods employing MTX selection conditions (Brown *et al.*, 1983; Tlsty *et al.*, 1984) because the fluorescence cell sorter is a much more sensitive means of detecting small changes in DHFR gene copy number than are the clonal selection methods.

7. The frequency of DHFR gene amplification can be increased dramatically by a variety of agents that inhibit reversibly DNA synthesis, including MTX (Tlsty *et al.*, 1982), hydroxyurea (Brown *et al.*, 1983), UV light and the carcinogen N-acetoxy N-acetylaminofluorene (Tlsty *et al.*, 1984). Although many of these agents produce "damage" in DNA, among their consequences is inhibition of DNA synthesis current findings in the laboratory suggest that it is the property of reversible inhibition of DNA synthesis that is important in the generation of gene amplification (see below).

8. Gene amplification is a common phenomenon in biology and is being reported increasingly in somatic cells. Amplification has been reported for a number of specific genes, including CAD (Wahl *et al.*, 1979), metallothionine (Beach & Palmiter, 1981), Hydroxymethylglutaryl CoA reductase (Chin *et al.*, 1982), adenosine deaminase (Yeung *et al.*, 1983), glutamine synthetase (Young & Ringold, 1983), ornithine decarboxylase (McConlogue *et al.*, 1984), and UMP synthetase (Kanalas & Suttle, 1984). In addition a number of investigators have reported a transport cross-resistance to a number of alkaloids with overproduction of one or more proteins and karyological and molecular evidence of gene amplification (Roninson *et al.*, 1984). There are some 10–15 other examples of gene amplification in somatic cells not listed here. Suffice it to indicate that such amplification events occur readily in somatic cells (see Schimke, 1984; Stark & Wahl, 1984).

*On the mechanism of gene amplification*

Studies in our laboratory have concentrated on the mechanism whereby hydroxyurea (HU) enhances the frequency of MTX resistance by virtue of gene amplification (Brown *et al.*, 1983). Mariani & Schimke (1984) have concluded that transient (6 h) inhibition of DNA synthesis, upon resumption in DNA synthesis, results in rereplication (overreplication) of the DNA replicated prior to inhibition of DNA synthesis in a subset of the cell population. This effect is observed only in cells that have progressed into the *S*-phase of the cell cycle. We have shown that the overreplication process involves not only the DHFR gene (which is replicated within the first hour of *S*), but virtually all of the DNA replicated prior to the onset of drug inhibition of DNA synthesis. More recently we have found (Johnston & Schimke, in preparation) that the critical variable in generating cells with additional DNA during recovery from inhibition of DNA synthesis with HU is the duration of inhibition of DNA replication. In the experiments of Mariani & Schimke (1984) the length of the mid *s*-phase block was 6 h. We now find that 6 h is a minimal time for demonstration of overreplication of DNA as indicated by the presence of cells with more than a G2 complement of DNA/cell as studied by flow cytometry and staining with either Hoechsts 33342 or chromomycin A3. The proportion of cells with greater than 2C DNA/cell increases with time of inhibition of DNA synthesis, when such cells are analyzed 24 h after removal of hydroxyurea. Hydroxyurea inhibits DNA synthesis but virtue of inhibition of ribonucleotide reductase. Similar results are obtained with the use of aphidicolin, an antibiotic specific for inhibition of DNA polymerase alpha of higher eukaryotes (Johnston & Schimke, in preparation).

We conclude that the initial event in selective gene amplification, e.g. of DHFR genes, is a non-specific overreplication of DNA. Inasmuch as DHFR is replicated in the first h of *S*-phase, it will have a high probability of being involved in the overreplication process. Various forms of recombination occur subsequently to generate chromosomal or extrachromosomal genes, and the *rare* cell with a productive recombination to generate a cell with increased DHFR enzyme activity will subsequently be selected. We wish to emphasize that the majority of the overreplicated DNA is unstable and will rapidly be lost in progeny that are not under selective pressure.

### *Overreplication of DNA and the generation of various of chromosomal abnormalities*

We are currently exploring the hypothesis that overreplication of DNA in a single cell cycle generates free-ended, double-stranded DNA that is highly recombinogenic (Schimke, 1984*b*). As long as such overreplicated DNA does not "invade" the sister chromatids in recombination, the structure of the newly replicated sister chromatids is intact. However, if such recombination occurs, it can result in inversions, sister chromatid exchanges or the generation of dicentric chromosomes, i.e. breakage–bridge fusion chromosomes. Inversions within HSR regions (Beidler *et al.*, 1980) as well as the presence of dicentric chromosomes (Fougere-Deschatrette, (1984) with amplified DHFR genes are common observations in karyotypes of cells with highly amplified DHFR genes. If only one of the two free DNA strands undergoes recombination into the chromosome, the result is a cell with a broken chromatid. Such cells cannot undergo a round of DNA synthesis subsequent to the cell cycle in which overreplication of DNA has occurred, and, hence, are essentially "dead".

Hill and Schimke (in preparation) have subjected mouse lymphoma cells (L5178Y) to a 6 h treatment with hydroxyurea and have analyzed such cells for DNA content and a spectrum of chromosomal aberrations as an immediate consequence of such treatment. As shown in Figure 1, cells subjected to transient inhibition of DNA synthesis result in a subset of cells 24 h later with increased DNA content/cell. When cells treated in this manner are analyzed by the fluorescence activated cell sorter, and sorted for such cells with greater than 2C DNA content, we observe that the major chromosomal abberations occur in the cells with the greater than 2C DNA content. A shown in Table I, cells with 2C DNA content have normal chromosomes. In contrast, 42% of chromosome spreads from cells with greater than 2C content are abnormal. The major aberrations include cells with fragmented chromosomes, some element of endoreduplication, or intact chromosomes with extrachromosomal DNA. Figure 2 shows four representative karyotypes showing (a) polyploidy, (b) intact chromo-

**Table I** Chromosomal changes in sorted cell populations after hydroxyurea treatment.

| | *% normal* | *% polyploid* | *% extra DNA* | *% fragmented* |
|---|---|---|---|---|
| No treatment | | | | |
| dull | 100 | 0 | 0 | 0 |
| bright | 100 | 0 | 0 | 0 |
| Hydroxyurea treatment | | | | |
| dull | 92 | 0 | 0 | 0 |
| bright | 42 | 18 | 10 | 30 |

Mouse L5178Y cells in continuous growth were treated for 6 h with 1.0 mM hydroxyurea, following which media was replaced with regular media. Colcemid was added 12 h later and cells were sorted for DNA content after staining with Hoechst's 33342 stain. "Dull" cells are those at the lower end of the fluorescence intensity. "Bright" cells are those at the high end of fluorescence intensity ([a]110 fluorescence units, See **Figure 1**). Two hundred metaphase spreads were examined for karyological abnormalities.

**Figure 1** Contour plots of L5178Y cell populations for DNA content analyzed by the fluorescence-activated cell sorter. Cells were stained for 1 h with Hoechst 33342 to determine DNA content per cells (*a*) control cells: 15.8% of cells had a fluorescence intensity of 110 units or greater; (*b*) cells pretreated with 10 mM hydroxyurea for 6 h and analyzed 24 h after removal of hydroxyurea: 35.2% of the cells had a fluorescence intensity of 110 units or greater. Contour plots are based on 10,000 cells; each line represents 20% of the population.

somes with extrachromosomal chromosomal DNA, (c) chromosomal fragmentation, (d) endoreduplication. What is particularly important is that the cells with the chromosomal aberrations occur *only* among that subset of the cell population with a DNA content/cell that is greater than 2C. Similar results (i.e. increased DNA content/cell and chromosomal aberrations) are also generated when cells are treated with aphidicolin or cells treated in mid *S*-phase with UV light and carcinogens (Tlsty & Sherwood, in preparation). The type of data lead us to suggest that the major rearrangements-alterations in genomes occur as a consequence of overreplication of DNA in a single cell cycle.

### Discussion

This review has provided evidence for heterogeneity of cultured cell populations when analyzed for the parameter of ability to grow under the selective conditions of MTX, a condition that frequently results from selection of cells with amplified DHFR genes. Further, our studies on the mechanism indicate that gene amplification results from a two-component process of initial overreplication of extensive amounts of the cell genome, followed by recombination events that allow expression and replication of the amplified genes, whether they be chromosomal or extrachromosomal.

Our results further suggest that a number of treatments of cultured cells that result in a transient inhibition of DNA synthesis *in cells in S-phase* results in overreplication of DNA. The selectable consequence of this process is drug resistance by virtue of gene amplification. However, we also find that the overreplication phenomenon results is a vast number of chromosomal aberrations-rearrangements. A majority of such chromosomal alterations have little long-term consequence, in that chromosomal fragmentation frequently results in cell death. Similarly, if the overreplicated DNA is extrachromosomal, it will be lost under non-selective conditions. Certain cells, however, will retain chromosomal rearrangements and/or differing degrees of polyploidy, i.e. aneuploidy, and there is likely to be random heterogeneity in those DNA sequences that undergo initial polyploidization-recombination events.

One can conceive of the process of tumour generation as (at least) a two step process. The first is the generation of cells that overcome normal growth regulation, i.e. they can now divide inappropriately. Such cells constitute a cancer. However it is well known that some cancers are "benign" and do not result in death, irrespective of their size or time of existence. In terms of solid tumours, according to Aver & Zetterberg (1984) those tumours that result in death are those that are aneuploid. Thus we suggest that a secondary

**Figure 2** Chromosomal aberrations induced by hydroxyurea. Metaphases were examined 24 h after removal of hydroxyurea and differentially stained sister chromatids were observed by FPG staining. (*a*) metaphase with normal chromosomes and a large amount of small, extrachromosomal DNA (arrow); (*b*) metaphase with multiple chromosome gaps and breaks; (*c*) and endoreduplicated metaphase with a large number of sister chromatid exchanges; (*d*), metaphase with fragmented chromosomes (arrow) showing a large number of sister chromatid exchanges.

process leading to lethal tumorigenesis is the generation of aneuploidy and the subsequent selection of certain cells from such populations with increased growth-metastatic potential. We suggest that this process results from loss of replication control (as opposed to growth control) such that overreplication of DNA occurs with the consequence of generation of aneuploidy. Among such consequences are, indeed, amplification of specific oncogenes, as observed both in continuous cell lines derived from tumours, as well as in tumour cell populations *per se* (see Schimke, 1984).

Our laboratory studies raise several questions concerning cancer and cancer treatment. Could not the heterogeneity observed in tumours result from the generation of aneuploidy, such heterogeneity itself resulting from imbalances in gene dosages, gene amplification, or recombinational inactivationactivation of specific genes? If there is indication that the answer to this question might be yes, then a major question follows: do not many of the cancer treatment modalities result in overreplication of DNA, the consequence of which is generation of chromosomal aberrations? Might such treatments convert relatively benign tumours into a state of aneuploidy and progression to more lethal form? Indeed, treatment of an experimental tumour with cancer chemotherapeutic agents has been shown to increase heterogeneity in DNA/cell (deVere White, 1983).

R.T.S. is American Cancer Society Research Professor of Biology. Supported by research grants from the National Institute of General Medical Sciences (GM 14931) and the National Cancer Institute (CA 13831).

### References

AVER, G. & ZETTERBERG, A. (1984). The prognostic significance of nuclear DNA content in malignant tumors of breast, prostate and cartilage. *Adv. Clin. Cytol.* **II**, Chapter 6.

BEACH, L.R. & PALMITER, R.D. (1981). Amplification of the metallothionein-1 gene in cadmium-resistant mouse cells. *Proc. Natl Acad. Sci.*, **78**, 2110.

BEIDLER, J.L. & SPENGLER, B.A. (1976). Metaphase chromosome anomaly: association with drug resistance and cell-specific products. *Science*, **191**, 185.

BEIDLER, J.L., MALERA, P.W. & SPENGLER, B.A. (1980). Specifically altered metaphase chromosomes in antifolate-resistant Chinese hamster cells that overproduce dihydrofolate reductase. *Cancer Genet. Cytogenet.*, **2**, 47.

BEVERLEY, S.M., CODERRE, J.A., SANTI, D.V. & SCHIMKE, R.T. (1984). Unstable DNA amplifications in methotrexate-resistant Leishmania consist of extrachromosomal circles which relocalize during stabilization. *Cell*, **38**, 431.

BROWN, P.E., TLSTY, T.D. & SCHIMKE, R.T. (1983). Enhancement of methotrexate resistance and dihydrofolate reductase gene amplification by treatment of mouse 3T6 cells with hydroxyurea. *Mol. Cell. Biol.*, **3**, 1097.

CARMAN, M.D., SCHOMAGEL, J.H., RIVEST, R.S. & 4 others. (1984). Resistance to methotrexate due to gene amplification in a patient with acute leukemia. *Clin. Oncol.*, **2**, 16.

CHIN, D.J., LUSKEY, K.L., ANDERSON, R.G.W., FAUST, J.R., GOLDSTEIN, J.L. & BROWN, M.S. (1982). Appearance of crystalloid endoplasmic reticulum in compactin-resistant Chinese hamster cells with a 500-fold increase in 3-hydroxy-3-methyglutaryl-coenzyme A reductase. *Proc. Natl Acad. Sci.*, **79**, 1185.

CURT, G.A., CARNEY, D.N., COWAN, K.H. & 6 others. (1983). Unstable methotrexate resistance in human small-cell carcinoma associated with double minute chromosomes. *N. Engl. J. Med.*, **208**, 199.

DE VERE WHITE, R., DEITCH, A.D. & OLSSON, C.A. (1983). Limitations of DNA histogram analysis by flow cytometry as method of predicting chemosensity in a rat renal cancer model. *Cancer Res.*, **43**, 604.

FOUGERE-DESCHATRETTE, C., SCHIMKE, R.T., WEIL, D. & WEISS, M.C. (1984). A study of chromosomal changes associated with amplified dihydrofolate reductase genes in rat hepatoma cells and their dedifferentiated variants. *J. Cell Biol.*, **99**, 497.

HABER, D.A., BEVERLEY, S.M., KIELY, M. & SCHIMKE, R.T. (1981). Properties of an altered dihydrofolate reductase encoded by amplified genes in cultured mouse fibroblasts. *J. Biol. Chem.*, **256**, 9501.

HAMKALO, B.A., FARNHAM, P.J., JOHNSTON, R. & SCHIMKE, R.T. (1985). Ultrastructural features of minute chromosomes in a methotrexate-resistant mouse 3T3 cell line. *Proc. Natl Acad. Sci.*, (in press).

HORNS, R.C., DOWER, W.J. & SCHIMKE, R.T. (1984). Gene amplification in a leukemic patient treated with methotrexate. *J. Clin. Oncol.*, **2**, 2.

JOHNSTON, R.N., BEVERLEY, S.M. & SCHIMKE, R.T. (1983). Rapid spontaneous dihydrofolate reductase gene amplification shown by fluorescence-activated cell sorting. *Proc. Natl Acad. Sci.*, **80**, 3711.

KANALAS, J.J. & SUTTLE, D.P. (1984). Amplification of the UMP synthetase gene and enzyme overproduction in pyrazofurin-resistant rat hepatoma cells. *J. Biol. Chem.*, **259**, 1848.

KAUFMAN, R.J. & SCHIMKE, R.T. (1981). Amplification and loss of dihydrofolate reductase genes in a Chinese hamster ovary cell line. *Mol. Cell. Biol.*, **1**, 1069.

KAUFMAN, R.J., BROWN, P.C. & SCHIMKE, R.T. (1981). Loss and stabilization of amplified dihydrofolate reductase genes in mouse sarcoma S-180 cell lines. *Mol. Cell. Biol.*, **1**, 1084.

MARIANI, B.D. & SCHIMKE, R.T. (1984). Gene amplification in a single cell cycle in Chinese hamster ovary cells. *J. Biol. Chem.*, **259**, 1091.

MCCONLOGUE, L., GUPTA, M., WU, L. & COFFINO, P. (1984). Molecular cloning and expression of the mouse ornithine decarboxylase gene. *Proc. Natl Acad. Sci.*, **81**, 540.

NUNBERG, J.H., KAUFMAN, R.F., SCHIMKE, R.T., URLAUB, G. & CHASIN, L.A. (1978). Amplified dihydrofolate reductase genes are localized to a homogeneously staining region of a single chromosome in a methotrexate-resistant Chinese hamster ovary cell line. *Proc. Natl Acad. Sci.*, **75**, 5553.

RONINSON, E.G., ABELSON, H.T., HOUSMAN, D.E., HOWELL, N. & VARSHAVSKY, A. (1984). Amplification of specific DNA sequences correlates with multidrug resistance in Chinese hamster cells. *Nature* **309**, 626.

SCHIMKE, R.T., BROWN, P.C., KAUFMAN, R.J., McGROGAN, M. & SLATE, D.L. (1981). Cyromosomal and extrachromosomal localizatio of amplified dihydrofolate reductase genes in cultured mammalian cells. *Cold Sprg. Harbour Symp. Quant. Biol.*, **45**, 785.

SCHIMKE, R.T. (1984*a*). Gene amplification in cultured animal cells. *Cell*, **37**, 705.

SCHIMKE, R.T. (1984*b*). Gene amplification, drug resistance, and cancer. *Cancer Res.*, **44**, 1735.

SIROTNAK, F.M., MOCCIO, D.M., KELLEHER, L.E. & GOUTAS, L.J. (1981). Relative frequency and kinetic properties of transport-defective phenotypes among methotrexate-resistant L1210 cell lines derived *in vivo*. *Cancer Res.*, **41**, 4447.

STARK, G.R. & WAHL, G.M. (1984). Gene amplification. *Ann. Rev. Biochem.*, **53**, 447.

TLSTY, T.D., BROWN, P.C., JOHNSTON, R. & SCHIMKE, R.T. (1982). Enhanced frequency of generation of methotrexate resistance and gene amplification in cultured mouse and hamster cell lines. In: *Gene Amplification*, (Ed. Schimke), New York: Cold Spring Harbor Laboratory, p. 231.

TLSTY, T.D., BROWN, P.E. & SCHIMKE, R.T. (1984). UV radiation facilitates methotrexate resistance and amplification of the dihydrofolate reductase gene in cultured 3T6 mouse cells. *Mol. Cell Biol.*, **4**, 1050.

TRENT, J.M., BUICK, R.N., OLSON, S., HORNS, R.C. & SCHIMKE, R.T. (1984). Cytologic evidence for gene amplification in methotrexate-resistant cells obtained from a patient with ovarian adenocarcinoma. *J. Clin. Oncol.*, **2**, 8.

WAHL, G.M., PADGETT, R.A. & STARK, G.R. (1979). Gene amplification causes overproduction of the first three enzymes of UMP synthesis in N-(phosphoacetyl-1-aspartate) resistant hamster cells. *J. Biol. Chem.*, **254**, 8679.

YEUNG, C.-Y., INGOLIA, D.E., BOBONIS, C. & 4 others. (1983). Selective overproduction of adenosine deaminase in cultured mouse cells. *J. Biol. Chem.*, **258**, 8338.

YOUNG, A.P. & RINGOLD, G.M. (1983). Mouse 3T6 cells that overproduce glutamine synthetase. *J. Biol. Chem.*, **258**, 11260.